# Localization of METTL16 at the Nuclear Periphery and the Nucleolus Is Cell Cycle-Specific and METTL16 Interacts with Several Nucleolar Proteins

**DOI:** 10.3390/life11070669

**Published:** 2021-07-08

**Authors:** Lenka Stixová, Denisa Komůrková, Alena Svobodová Kovaříková, Paolo Fagherazzi, Eva Bártová

**Affiliations:** 1 Institute of Biophysics of the Czech Academy of Sciences, Královopolská 135, 612 65 Brno, Czech Republic; komurkova@ibp.cz (D.K.); alenakovarikova@ibp.cz (A.S.K.); fagher@ibp.cz (P.F.); 2 Faculty of Science, Masaryk University, Kamenice 753/5, 601 77 Brno, Czech Republic

**Keywords:** METTL16, nucleolus, cell cycle, rDNA, epitranscriptome

## Abstract

METTL16 methyltransferase is responsible for the methylation of N^6^-adenosine (m^6^A) in several RNAs. In mouse cells, we showed that the nuclear distribution of METTL16 is cell cycle-specific. In the G1/S phases, METTL16 accumulates to the nucleolus, while in the G2 phase, the level of METTL16 increases in the nucleoplasm. In metaphase and anaphase, there is a very low pool of the METTL16 protein, but in telophase, residual METTL16 appears to be associated with the newly formed nuclear lamina. In A-type lamin-depleted cells, we observed a reduction of METTL16 when compared with the wild-type counterpart. However, METTL16 does not interact with A-type and B-type lamins, but interacts with Lamin B Receptor (LBR) and Lap2α. Additionally, Lap2α depletion caused METTL16 downregulation in the nuclear pool. Furthermore, METTL16 interacted with DDB2, a key protein of the nucleotide excision repair (NER), and also with nucleolar proteins, including TCOF, NOLC1, and UBF1/2, but not fibrillarin. From this view, the METTL16 protein may also regulate the transcription of ribosomal genes because we observed that the high level of m^6^A in 18S rRNA appeared in cells with upregulated METTL16.

## 1. Introduction

N^6^-methyladenosine (m^6^A) is the most prevalent and intensively studied RNA modification. This post-transcriptional event regulates multiple processes, including pre-mRNA splicing, stability, and subsequent translation. Most m^6^A marks in mRNA are mediated via a methylome complex that contains methyltransferases METTL3/METTL14 (methyltransferase-like 3/methyltransferase-like 14) [1]. Moreover, METTL16 (methyltransferase-like 16; METT10D) is the human SAM-dependent m^6^A methyltransferase, which works similarly to other recently discovered methyltransferases, including CAPAM, METTL5/TRMT112, and ZCCHC4 [2,3,4,5]. Apart from installing m^6^A modification by writers such as METTL3/METTL14 and METTL16 enzymes, demethylation processes can be activated. In this case, two main erasers are involved: FTO (fat mass and obesity-associated protein) and ALKBH5 (ALKB homolog 5) [6,7]. These proteins are responsible for oxidation-based demethylation of m^6^A in distinct types of RNAs. The functions of “writers” and “erasers” indicate that methylation processes in RNAs are dynamic and reversible, which is essential for the functional properties of RNAs. It is well-known that epitranscriptomic features, including methylation of RNAs, regulate the expression of specific genes that are responsible for many cellular processes, including cell differentiation, cell fate, or embryonic development [8,9]. For instance, Ruszokovszka [10] demonstrated that the function of human METTL16 protein is essential for N^6^-methyladenosine (m^6^A) appearance in RNA that originates from genes regulating apoptosis as well as cell proliferation in C. ellegans. Moreover, a pronounced interaction between METTL16/MALAT1 (metastasis-associated lung adenocarcinoma transcript 1) is frequent in tumor cells [11].

The METTL16 protein belongs to the class I methyltransferase family. METTL16 is responsible for N^6^ methylation of adenosine 43 on the U6 snRNA and induces m^6^A modification in the MAT2A mRNA encoding S-adenosylmethionine (SAM) synthetase [5,12,13]. Concurrently, METTL16 binds to other non-coding RNAs, long non-coding RNAs, and several intronic sequences in pre-mRNAs [5,12,13,14,15]. Although tRNAs and ribosomal RNAs (rRNAs) are the most highly modified RNA species, the deposition of m^6^A in rRNA and tRNA is less well-understood and needs to be explored [16]. It is known that METTL16 exists as a homodimer, but homodimerization is not essential for METTL16 binding to RNA and its catalytic activity [17]. For example, monomeric METTL16 recognizes and methylates U6 and MAT2A RNA in vitro [12], while the METTL16 homodimer can interact with the MALAT1 RNA triple helix. This observation suggests different recognition mechanisms for distinct RNA substrates [16]. The structure of METTL16 is similar to the METTL3 enzyme, but both proteins have some unique elements, such as the unique αB helix in the Rossmann fold [11]. Interestingly, METTL16 acts as an enzyme considered both a writer and a reader. For its methylation activity as a writer, METTL16 requires the UACAGAGAA nonamer sequence and specific stem-loop RNA structure [12,16]. As a writer, METTL16 methylates the MAT2A mRNA in the presence of SAM, which leads to intron retention and subsequent nuclear degradation [12]. METTL16 as a reader contributes to the splicing of SAM synthetase transcript [10,12]. Recent findings suggest an essential role of METTL16 in mRNA stability and splicing. Moreover, Brown et al. demonstrated its interactions with rRNA [14], and Warda explained METTL16 enrichment by non-specific cross-linking arising from the presence of METTL16 in the nucleolus [5]. Additionally, it is known that METTL16 interacts with the MALAT1 triple helix associated with proteins, such as DYNLL1 and NPM, DHX9, ILF3, or ILF2 [14,18]. Surprisingly, the interaction of these components (METTL16 and NPM) was not RNA-dependent; therefore, METTL16 likely plays a role in other non-specified nuclear processes [14]. This observation indicates that the proper physiological role of the METTL16 protein in RNA processing and function is not as fully elucidated as the precise mechanism of METTL16 function in DNA damage response (DDR). In the latter case, Svobodova Kovarikova et al. [19] showed recruitment of the METTL16 protein to locally induced DNA lesions at the later steps of DDR when m^6^A in RNA disappears from damaged chromatin. Xiang et al. (2017) [8] revealed that m^6^A RNA is recruited to UVA-induced DNA lesions immediately after irradiation and remains stable at the lesions up to 5 min after genome injury. This process was dependent on the function of METTL3 and METTL14 methyltransferases.

In this work, we were inspired by these observations; therefore, we analyzed how DNA damage and cell cycle phases affect the levels and distribution of the METTL16 protein. Furthermore, due to the appearance of METTL16 in the nucleolus, we studied the regulatory role of METTL16 in the post-transcription processing of ribosomal genes. Additionally, due to the high density of the METTL16 protein at the nuclear periphery in mouse cells, we studied how A- and B-type lamins and lamin-associated proteins contribute to METTL16 function and distribution in the cell nucleus.

## 2. Materials and Methods

### 2.1. Cell Cultivation and Treatment

GOWT1 mouse embryonic stem cells (mESCs) stably expressing exogenous Oct4 (a generous gift from Hitoshi Niwa, Laboratory for Pluripotent Stem Cell Studies, RIKEN Center for Developmental Biology, Kober, Japan) were cultivated in Dulbecco’s modified Eagle medium (DMEM, #P04-03550, PAN Biotech, GmbH, Aidenbach, Germany) containing 4.5 g/L glucose and L-glutamine, supplemented with 15% fetal calf serum (tested for ESCs), 1 × non-essential amino acids (final concentration 0.1 mM, Invitrogen, CZ), sodium pyruvate (1 mM), MTG (monothioglycerol; 100 µM), 10,000 IU/mL penicillin, 10,000 µg/mL streptomycin, and leukemia inhibitory factor (LIF, final concentration 10 ng/mL, ESG1107, Merck Millipore, Darmstadt, Germany). Lap2α (wt) and Lap2α (dn) mouse embryonic fibroblasts (MEFs) [20] originated from the laboratory of Prof. Roland Foisner (Max F. Perutz Laboratories in Vienna, Austria) and were cultivated in Dulbecco’s modified Eagle’s medium (DMEM, Sigma-Aldrich, Prague, Czech Republic) with 10% fetal bovine serum, 1 × non-essential amino acids (final concentration 0.1 mM, Invitrogen, CZ), and 10,000 IU/mL penicillin and 10,000 µg/mL streptomycin. Wild-type (wt) and *lmna* double knock-out (dn) mouse embryonic fibroblasts (MEFs; a gift from Dr. Teresa Sullivan and Dr. Collin L. Stewart from the Institute of Medical Biology, Singapore), HeLa (ATCC^®^ CCL-2™), MCF7, MCF7 LBR(-) [21] (a generous gift from Dr. Emilie Lukasova, Department of Molecular Epigenetics, Institute of Biophysics of the Czech Acad. Sci. in Brno, Czech Republic), HaCaT (purchased from ATTC, Manassas, VA, USA, or the European Tissue Culture Collection, UK), and HT-29 (ATCC) cells were cultivated in Dulbecco’s modified Eagle’s medium supplemented with 10% fetal calf serum (FCS) and appropriate antibiotics at 37 °C in a humidified atmosphere containing 5% CO_2_. 

For cell transfection, the cells were cultivated on glass-bottomed tissue culture dishes and transfected with 2 μg plasmid DNA encoding GFP-UBF (#17656; Addgene, Cambridge, MA, USA). Transfections were performed using METAFECTENE^TM^PRO reagent (#T040-2.0, Biontex Laboratories GmbH, Germany).

For experiments, 24 h after seeding, the cells were irradiated with either UVA light (model GESP-15, 15 W; UVC Servis, Prague, Czech Republic) or a UVC lamp (model TUV 30 W T8; Philips, The Netherlands). The cells were harvested at approximately 70% confluence 1 h after irradiation. Cells were treated with 0.5 μg/mL Actinomycin D (#A9415, Sigma-Aldrich, St. Louis, MO, USA) for 1 h.

### 2.2. Immunofluorescence, Analysis of Nuclear Protein Distribution, and Confocal Microscopy

For immunofluorescence staining, the cells were fixed in 4% paraformaldehyde for 20 min at room temperature (RT), permeabilized with 0.3% Triton X-100 (Merck) for 10 min, and 0.1% saponin (Sigma-Aldrich) for 12 min, and washed twice in phosphate-buffered saline (PBS) for 10 min. Bovine serum albumin (Merck) (1% dissolved in PBS-Tween 20 (0.1%) was used as a blocking solution. The following antibodies were used at a 1:100 dilution: anti-METTL16 (#HPA020352, Atlas Antibodies, Bromma, Sweden) and the METTL16 antibody anti-MET10 (#orb 27848, Biorbyt, Cambridge, UK), anti-H3S10 (#ab14955, Abcam, Cambridge, UK), anti-Fibrillarin (#ab4566, Abcam), anti-Lamin A/C (#SAB4200236, Sigma-Aldrich), anti-Lamin B (#ab8982, Abcam), anti-Lamin B Receptor (#ab32535, Abcam), anti-Lamin B Receptor (#NBP2-59947, Novus Biologicals, Bio-Techne Ltd., Abingdon, UK). Secondary antibodies used were: Alexa 488-conjugated goat anti-rabbit (#ab150077, Abcam), Alexa 594-conjugated goat anti-rabbit (#A11037, ThermoFisher Scientific, Waltham, MA, USA), Alexa 488-conjugated goat anti-mouse (#A11029, ThermoFisher Scientific), and Alexa Fluor^®^ 594 anti-mouse (#A-11032, ThermoFisher Scientific). As a negative control, we used samples incubated without primary antibodies. For visualization of cell nuclei, we used 4′,6-diamidino-2-phenylindole (DAPI; Merck). The samples were mounted with Vectashield Mounting Medium (#H-1000, Vector Laboratories, Burlingame, CA, USA).

Confocal microscopy was performed by the use of a Leica TCS SP8 X SMD confocal microscope (Leica Microsystems GmbH, Wetzlar, Germany), supplemented with a PicoHarp 300 module (PicoQuant GmbH, Berlin, Germany) and HyD SMD detectors. Image acquisition was performed using a white light laser (WLL; wavelengths of 470–670 nm in 1-nm increments) with the following parameters: 1024 × 1024 pixels, 400 Hz, bidirectional scanning mode, zoom 5–9. The fluorescence intensity (FI) from specific subcellular regions of individual cells was analyzed using LAS AF Lite 4 software.

### 2.3. Coimmunoprecipitation and Western Blot Analyses

For immunoprecipitation (IP), the cells were lysed in Pierce™ IP Lysis Buffer (#87787, ThermoFisher Scientific) supplemented with aprotinine (1 µg/mL) and PMSF (1 µg/mL) for 5 min on ice and centrifuged at 13,000× *g* for 10 min at 4 °C. Immunoprecipitation was performed using the Catch and Release v2.0 Reversible Immunoprecipitation Kit according to the manufacturer’s protocol (no. 17-500, Merck Millipore, Billerica, MA, USA). Briefly, the column resin was washed using the provided buffer. The following ingredients were added to each column: 500 μg of the appropriate cell lysate, 5 μL of anti-METTL16 antibody (#HPA020352, Atlas Antibodies), or control IgG antibody (anti-rabbit IgG (A-4914, Sigma Aldrich) and an Antibody Capture Affinity Ligand. The final volume was adjusted to 500 μL using the provided 1 × wash buffer. The columns were incubated overnight on a rotator at 4 °C. The following day, the columns were washed with the provided wash buffer; immunoprecipitated proteins were eluted in denaturing buffer. We used 10 μL of denaturation buffer that was subjected to SDS–polyacrylamide gel electrophoresis, and 10 μg of total protein lysate was used as input.

Western blotting was performed following [22]. The proteins were separated by SDS–polyacrylamide gel electrophoresis (SDS–PAGE; 10% polyacrylamide) and transferred to polyvinylidene difluoride (PVDF) membranes. The membranes were blocked with 2% nonfat milk for 1 h and then immunoblotted with the following antibodies overnight at 4 °C: anti-METTL16 antibody (#HPA020352, Atlas Antibodies), anti-α-tubulin (#ab80779 Abcam), anti-NOLC1 (#A5899, ABclonal, Woburn, MA, USA), TCOF (#A65212, ABclonal), anti-UBF1 (#ab75781, Abcam), anti-Fibrillarin (#ab4566, Abcam), anti-Lamin B (#ab8982, Abcam), anti-Lamin B Receptor (#ab32535, Abcam), anti-Lap2α (#ab5162, Abcam). As secondary antibodies, we used goat anti-rabbit IgG (#AP307P, Merck, Prague, Czech Republic; 1:2000), anti-mouse IgG (#A9044, Sigma-Aldrich, Prague, Czech Republic; 1:2000), and goat anti-mouse IgG1 (#ab97240, Abcam). The detection of proteins was performed using ECL (#RPN2232, Amersham Biosciences, Amersham, UK) chemiluminescence reagent analyzed using a LAS-3000 (Fujifilm, Tokyo, Japan).

### 2.4. RNA-Binding Protein Immunoprecipitation Combined with Quantitative Polymerase Chain Reaction (RIP-qPCR)

RNA-binding protein immunoprecipitation (RIP) was performed according to the manufacturer’s protocol (Magna RIP^TM^ RNA-Binding Protein Immunoprecipitation Kit, Millipore, Darmstadt, Germany). For analysis, we used 20 × 10^6^ cells per RIP, determined using a TC10™ automated cell counter (Bio-Rad, Prague, Czech Republic). RNA-binding protein–RNA complexes were incubated overnight at 4 °C with 10 μL of anti-m^6^A (#202111, Synaptic Systems, Goettingen, Germany) or 5 µL Normal Mouse IgG (as a negative control, background, included in the kit).

The polymerase chain reaction (PCR) was performed as previously described [23], and the following primers were used: rRNA 28S fwd 5′-CCAAATGCCTCGTCATCTAA-3′ and rev 5′-CTCAACAGGGTCTTCTTTCC-3′ [24,25] and rRNA 18S fwd 5′-GTAACCCGTTGAACCCCATT-3′ and rev 5′-CCATCCAATCGGTAGTAGCG-3′ [22]. For real-time PCR, we used the FastStart Universal SYBR Green Master (Rox) (Roche, Mannheim, Germany). We performed 40 cycles, and denaturation was at 94 °C for 1 min. Primers were annealed at 59 °C for 30 s. The extension temperature was 72 °C for 30 s, and finally, we carried out an extension at 72 °C for 5 min. The PCR reaction was performed using the cycler QuantStudio™ 5 Real-Time PCR System (Applied Biosystems™, Thermo Fisher Scientific, Waltham, MA, USA). Samples with mouse IgG served as negative controls of immunoprecipitation, and samples without DNA were considered as a negative control for PCR reaction. 

### 2.5. Statistical Analysis

Statistical analysis was performed using Sigma Plot 13.0 (Jandel Scientific, San Rafael, CA, USA). When the normality test passed, then the Student’s test was calculated. If the normality test (Shapiro–Wilk) failed (*p* < 0.050), then the Mann–Whitney rank-sum was applied. The statistical significance is shown in the graphs.

## 3. Results

### 3.1. Cellular Localization of the METTL16 Protein in Different Cell Lines

**Figure 1:**
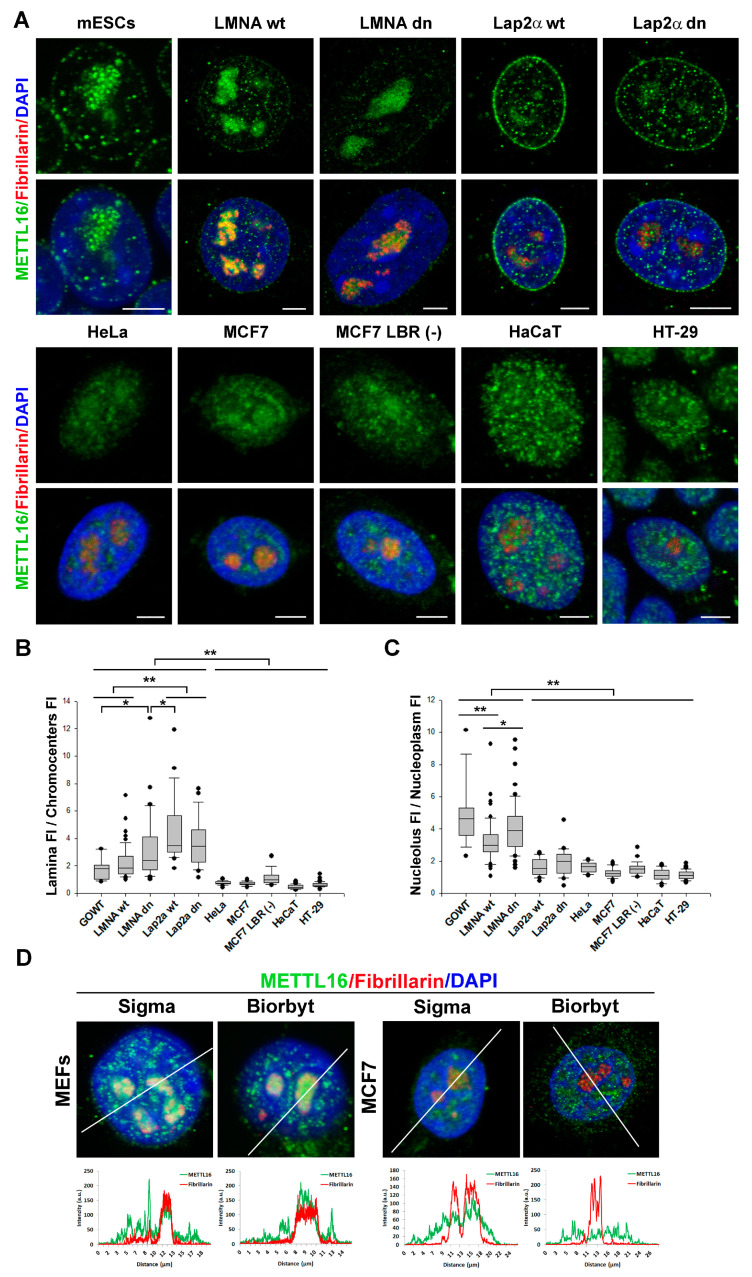
The subcellular distribution of the METTL16 protein in different cell lines. (**A**) Immunofluorescence shows METTL16 nuclear distribution in selected cell lines (GOWT, MEFs LMNA wt, and LMNA dn, mouse dermal fibroblasts LAP2α wt and LAP2α dn, HeLa, MCF7, MCF7 LBR (-), HaCaT, and HT-29 cells). Staining was performed with an antibody against METTL16 (green) and fibrillarin (red). DNA was stained with DAPI (4′,6-diamidino-2-phenylindole, blue). Scale bars show 5 µm. (**B**) Quantitative analysis of METTL16 immunofluorescence. Boxplot depicting the ratios in the fluorescence intensity (FI) of Alexa fluor 488-stained METTL16 in the nuclear lamina to FI of METTL16 in the DAPI-stained chromocenters in different cell lines. Panel (**C**) shows the ratios of the Alexa fluor 488-stained METTL16 (FI) in the nucleolus to FI of METTL16 in the nucleoplasm. Box plots show median values with upper and lower quartiles; error bars represent 10th and 90th percentiles (from left to right, the number of measurements was: *n* = 25, 55, 52, 25, 26, 26, 34, 27, 33, 48). Statistical analysis was performed using the Sigma Plot 13.0 software (Jandel Scientific, San Rafael, CA, USA). Because the normality test (Shapiro–Wilk) failed, the Mann–Whitney rank-sum test was applied. This test confirmed the differences in the median values of fluorescence intensity for ∗ α ≤ 0.01, ∗∗ α ≤ 0.001. (**D**) Panels show the comparison of METTL16 cellular distribution studied by two specific antibodies against METTL16. Analysis by immunofluorescence and FI quantification was performed in MEFs and human MCF7 cells.

Immunofluorescence was used to study the cellular distribution of the METTL16 protein in different cell lines. We studied mouse cells, including mouse embryonic stem cells (mESCs GOWT), mouse embryonic fibroblasts (MEFs; A-type lamin wild-type (wt), and A-type lamin double null; dn), mouse dermal fibroblasts (LAP2α wt and LAP2α double null), and human carcinoma cells MCF7, HeLa, HT29, or keratinocytes HaCaT. In mouse cells, a high level of METTL16 colocalizing with nuclear lamina was found. At the nuclear periphery, we observed the highest density of METTL16 in LAP2α wt and LAP2α dn cells (Figure 1A,B). In all cellular models studied, we also found that METTL16 was accumulated in the fibrillarin-positive region of nucleoli (Figure 1A,C). Among the cell types analyzed, the highest density of the METTL16 protein was found in the nucleoli of GOWT mESCs (Figure 1A,C). We analyzed METTL16 in the nucleolus of selected mouse and human cell lines using two specific antibodies. Although there were some differences between mouse cells and human cell line MCF7, the presence of METTL16 in the nucleolus was found in the cells of both mouse and human origin (Figure 1D). Cell type-specific distribution of METTL16 in human cells is also documented at https://www.proteinatlas.org/ENSG00000127804-METTL16/cell (accessed on 7 July 2021), showing preferential localization of METTL16 in the cytoplasm of many human cells, but U-251 MG cells also show localization in the nucleoplasm. Nance et al. [26] documented 50% of METTL16 localized in the cytoplasm of human cells, including HEK293T and HeLa. Therefore, it is evident that the distribution profile of the METTL16 protein is cell type-specific.

### 3.2. Cellular Localization of METTL16 in Individual Cell Cycle Phases

**Figure 2:**
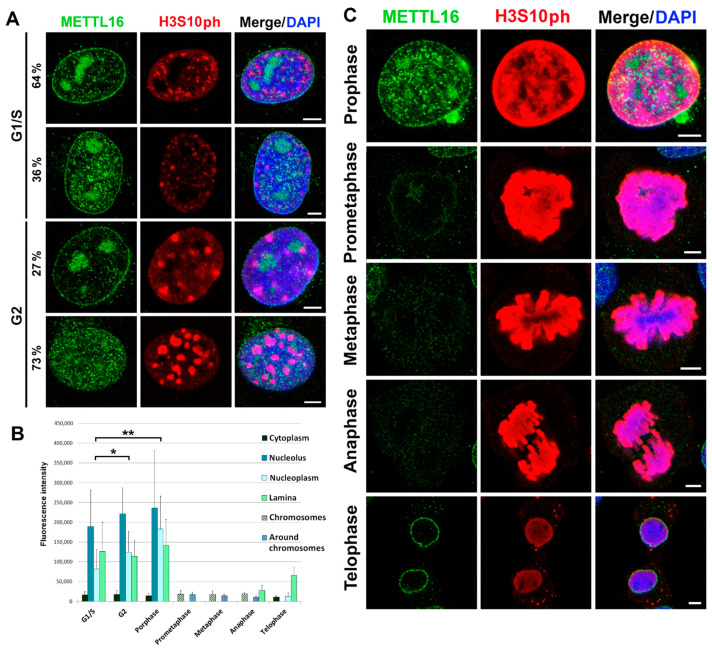
Cellular localization of METTL16 in individual cell cycle phases. Double immunofluorescence labeling of METTL16 (green) and phosphorylation of histone H3, at serine 10 (H3S10ph) (red) of MEFs in (**A**) interphase. Distribution profile of H3S10ph was selected due to its specificity in G1/S (tiny foci) and G2 phases (robust foci) of the cell cycle. (**B**) Quantification of METTL16 fluorescence intensity in the cytoplasm, nucleolus, nucleoplasm, and nuclear lamina during the cell cycle (error bars show the standard deviation for the number of measurements were; *n* = 30, 25, 10, 10, 10, 10, 20). Mann–Whitney rank-sum test showed statistically significant differences at ∗ α ≤ 0.05, ∗∗ α ≤ 0.01. (**C**) Distribution pattern of METTL16 (green) and H3S10ph (red), studied by immunofluorescence, in mitotic cells. Scale bars: 5 μm.

We further analyzed the subcellular distribution of the METTL16 protein in MEFs during the cell cycle. Immunofluorescence analysis showed that the nuclear distribution of METTL16 is different in individual phases of the cell cycle. In the G1/S phases, METTL16 accumulates predominantly in the nucleolus. In these cell cycle phases, in the majority of cells, we found a lower level of METTL16 in the nucleoplasm, while the G2 phase was characterized by an increased METTL16 level in the nucleoplasm (Figure 2A,B). Interestingly, when compared to interphase, especially prometaphase of mitosis, metaphase, and anaphase were characterized by a significantly reduced level or even the disappearance of METTL16 (Figure 2B,C). However, the last phases of anaphase and telophase were characterized by significant relocalization of the residual METTL16 protein on the nuclear periphery. At this cell cycle stage, METTL16 protein becomes a component of the nuclear lamina, especially in mouse cells (Figure 2C).

In summary, our data suggest that, especially in mouse cells, the nuclear distribution of METTL16 is cell cycle-specific. In the G1/S phase, METTL16 is functional, especially in the nucleolus, while in the G2 phase of the cell cycle, METTL16 is located in the whole nucleoplasm. From prometaphase to anaphase, there is a low nuclear pool of the METTL16 protein, but in telophase, METTL16 likely contributes to nuclear lamina formation, maintenance or may interact with other lamina-associated proteins.

### 3.3. UVA and UVC Irradiation Upregulate METTL16

**Figure 3:**
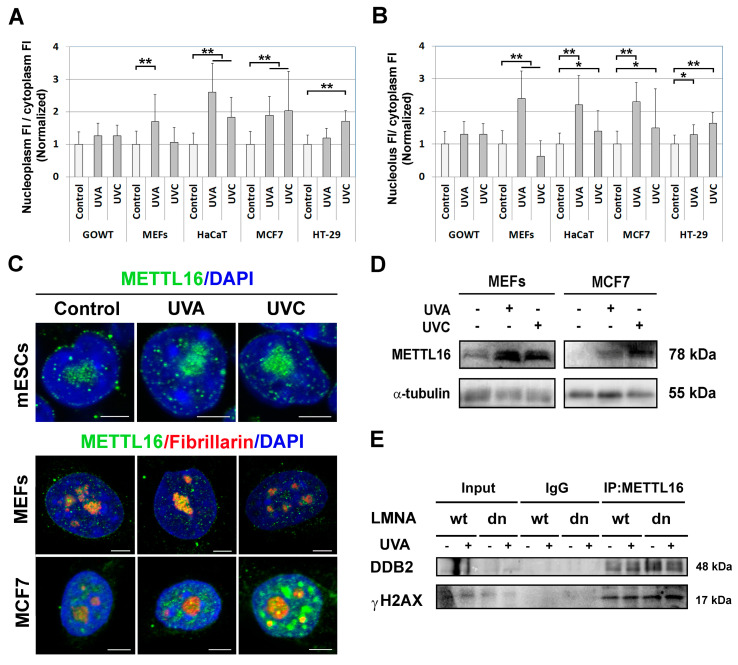
The changes in METTL16 subcellular distribution after UVA and UVC irradiation. Quantitative immunofluorescence analysis of METTL16 distribution was performed in selected cell lines. (**A**) The graph depicts the normalized ratios of METTL16 fluorescence intensity (FI) values in the nucleoplasm to METTL16 FI in the cytoplasm. Panel (**B**) shows normalized ratios of METTL16 FI in the nucleolus to METTL16 FI in the cytoplasm (from left to right the number of measurements were: *n* = 25, 20, 23, 30, 30, 31, 34, 20, 20, 34, 20, 20, 48, 32, 20). Error bars represent standard deviations (S.D.). Statistical analysis was performed using Mann–Whitney rank-sum test. Statistically significant differences are shown at ∗ α ≤ 0.05, ∗∗ α ≤ 0.001. (**C**) Immunofluorescence labeling of METTL16 (green) in mESCs and dual labeling of METTL16 (green) with fibrillarin (red) in MEFs and MCF7 cells. (**D**) Western blot analysis shows the level of the METTL16 protein in MEFs and MCF7 cells exposed to UVA and UVC irradiation (original data see in Supplementary Figure S1). Data were normalized to the total protein levels and to the level of α-tubulin. (**E**) Co-IP and Western blots of METTL16 with the DDB2 protein or METTL16 with γH2AX, studied in wt MEFs and LMNA dn cells.

To study the function of METTL16 in the DNA damage response, the cells were irradiated by UVA and UVC light as whole populations. Using immunofluorescence, we analyzed the distribution profile of METTL16 in selected cell lines exposed for 10 min to UVA and UVC light. The results shown in Figure 3A indicate that the level of METTL16 is increasing 1 h after irradiation in both the nucleoplasm and the nucleolus. The increase in the METTL16 nuclear pool after both UVA and UVC irradiation correlated with data shown by Western blot analysis in MEFs and MCF7 cells (Figure 3D). From the view of DNA repair processes, we observed that METTL16 interacts with DDB2, which is a key player in the nucleotide excision repair (NER) mechanism (Figure 3E). Moreover, we found that METTL16 interacts with phosphorylated histone H2AX (γH2AX), which is a marker of double-strand breaks (DSBs). It is evident that UV light induces a mixture of distinct DNA lesions, preferentially cyclobutane pyrimidine dimer (CPDs), recognized by the NER mechanism, but secondary DNA lesions are DSBs. In this case, it seems likely that METTL16 participates in this multifactorial DNA repair process, activated by UV light.

### 3.4. METTL16 Response to Irradiation Is Linked to A-Type Lamin and Lap2α Functions

**Figure 4:**
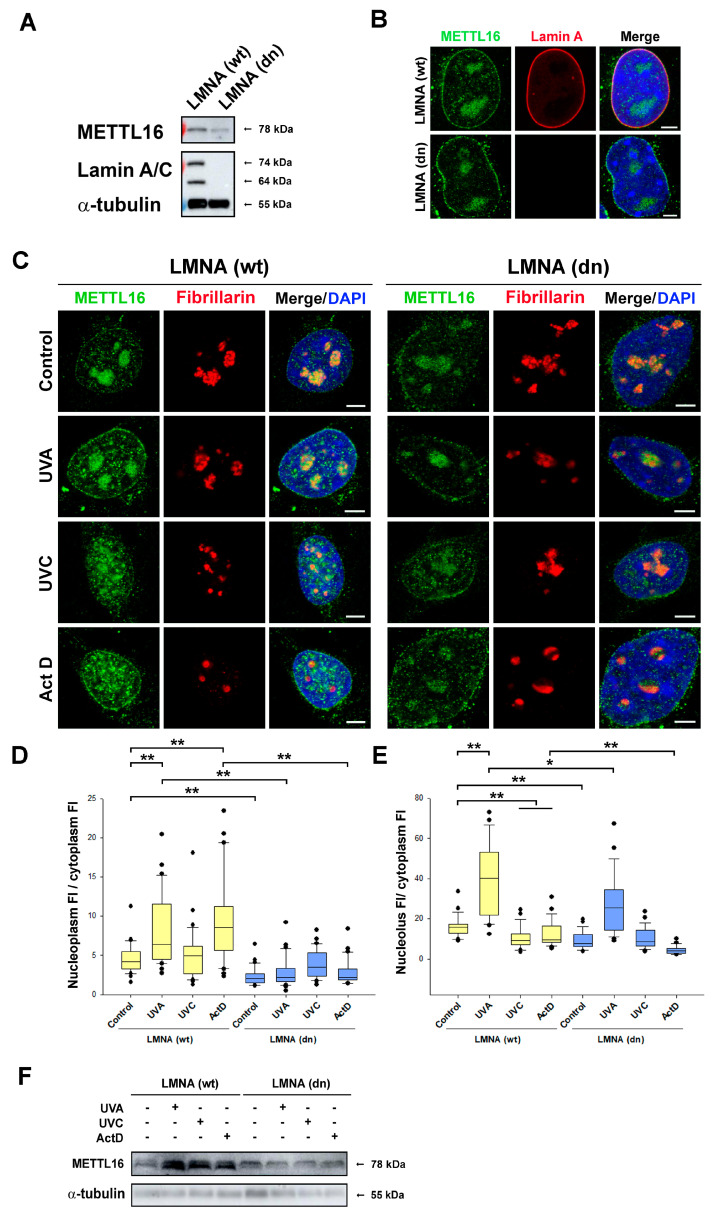
Effect of UVA and UVC irradiation and Actinomycin D treatment on METTL16 localization in LMNA wt MEFs and LMNA double null (dn) cells. (**A**) Western blot analysis of Lamin A/C and METTL16 protein; α-tubulin was used as the loading control. Analysis was performed in LMNA wt MEFs and LMNA dn cells. (**B**) Immunofluorescence of wt MEFs and LMNA dn cells stained with antibody against METTL16 (green) and Lamin A (red). DNA was stained with DAPI (blue). Scale bars show 5 µm. (**C**) Representative immunofluorescence images of METTL16 (green) and nucleolar protein fibrillarin (red). The cells were irradiated by UVA, UVC light, or treated by ActD. Analysis was performed 1 h after the treatment. DNA was stained with DAPI (blue). Scale bars show 5 µm. (**D**,**E**) Quantitative immunofluorescence analysis of METTL16 distribution in wt MEFs and LMNA dn cells. (**D**) Boxplot depicts the ratios of the METTL16 fluorescence intensity (FI) values in the nucleoplasm to METTL16 FI in the cytoplasm. Panel (**E**) shows the ratios of the METTL16 FI in the nucleolus to METTL16 FI in the cytoplasm. All box plots show median values with upper and lower quartiles; error bars represent 10th and 90th percentiles. (n_1_ − n_8_ = 30). Statistical analysis was performed using the Mann–Whitney rank-sum test. Statistically significant differences are shown at ∗ α ≤ 0.01, ∗∗ α ≤ 0.001. (**F**) Western blot analysis of the METTL16 protein in LMNA wt MEFs and LMNA dn cells exposed to UV irradiation or treated by Actinomycin D. Western blot data were normalized to the total protein levels and to the level of α-tubulin.

Due to the fact that we observed the METTL16 protein as a component of the nuclear lamina during interphase and telophase (Figure 2A,B), we addressed the question of whether depletion of the *lmna* gene can affect the nuclear distribution pattern and function of the METTL16 protein in cells exposed to UV irradiation or treated by Actinomycin D, an inhibitor of RNA polymerase I regulating the transcription of ribosomal genes in the nucleolus. In these experiments, we studied LMNA-depleted (LMNA dn) mouse embryonic fibroblasts (MEFs), characterized by an absence of A-type lamins (Figure 4A,B). In comparison with the wild-type (wt) cells, we observed a lower level of the METTL16 protein in LMNA dn cells, as shown by the quantification of immunofluorescence data and by Western blots (Figure 4C–F). In wt MEFs, UVA irradiation-induced an increased level of METTL16 in the nucleoplasm and in the nucleolus when compared to the cytoplasm (Figure 4D,E). Additionally, Actinomycin D (ActD) treatment increased the level of METTL16 in the nucleoplasm compared to the cytoplasm; however, it slightly reduced the nucleolar level of METTL16 (Figure 4D,E). These results show that in the pool of METTL16, its nuclear distribution profile can be affected by UV irradiation, inhibition of RNA pol I, and A-type lamin deficiency (Figure 4D–F). In mouse ESCs, it was observed by other authors that METTL16 has two isoforms produced by alternative splicing (https://www.uniprot.org/uniprot/Q9CQG2 (accessed on 7 July 2021)); we also observed by Western blot a double band indicating the existence of two METTL16 isoforms. This phenomenon was shown in LMNA wt cells, but in LMNA dn cells, this double band was barely detectable (Figure 4F).

**Figure 5:**
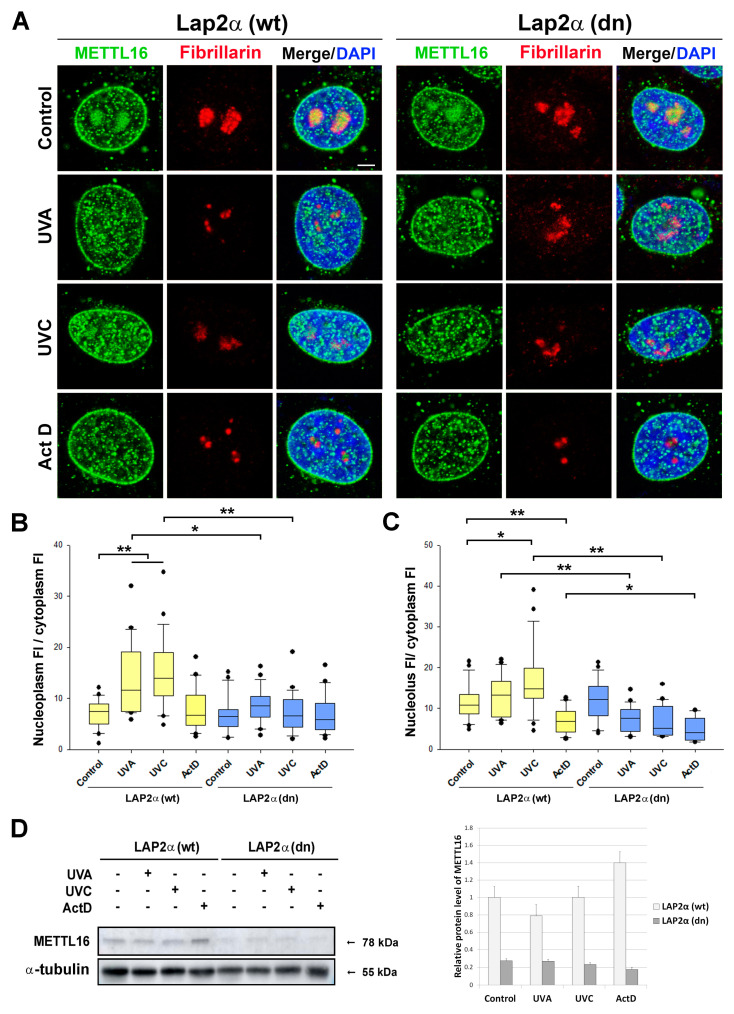
Effect of UVA and UVC irradiation and Actinomycin D treatment on METTL16 localization in mouse dermal fibroblasts (LAP2α wt and LAP2α dn). (**A**) Representative immunofluorescence images of METTL16 (green) and fibrillarin (red). The cells were irradiated by UVA, UVC irradiation, or treated by ActD and allowed to recover for 1 h after treatments. DNA was stained with DAPI (blue). Scale bar, 5 µm. Quantitative immunofluorescence analysis of METTL16 distribution. (**B**) Boxplot shows the ratios of the METTL16 fluorescence intensity (FI) values in the nucleoplasm to METTL16 FI in the cytoplasm. Panel (**C**) shows ratios of the METTL16 FI in the nucleolus to METTL16 FI in the cytoplasm. All box plots document median values with upper and lower quartiles; error bars represent 10th and 90th percentiles. (n_1_ − n_8_ = 25). Statistical analysis was performed using the Mann–Whitney rank-sum test for ∗ α ≤ 0.05, ∗∗ α ≤ 0.001. (**D**) Western blot analysis of METTL16 protein in LAP2α wt and LAP2α dn cells irradiated by UV light or treated by ActD.

The level and the nuclear distribution of the METTL16 protein were also studied in LAP2α wt and LAP2α dn mouse dermal fibroblasts (Figure 5A–D). In wt cells, we observed an increased level of METTL16 in the cell nucleoplasm and nucleolus after irradiation and a decreased level of METTL16 in the nucleolus after Actinomycin D treatment (Figure 5B,C). The effect of UVA and UVC irradiation on the level of METTL16 in the nucleoplasm and nucleolus was not observed in Lap2α-deficient cells. A reduced pool of METTL16 was also detected by Western blot analysis in LAP2α dn cells when compared to their wild-type counterparts (Figure 5D).

### 3.5. Relationship between METTL16 and Nucleolar Proteins Essential for rRNA Synthesis

**Figure 6:**
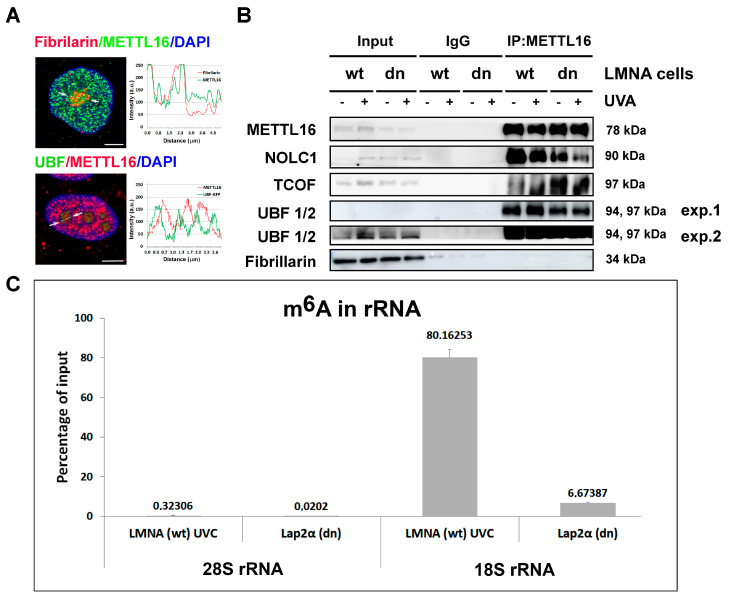
Localization of METTL16 in the nucleolus. (**A**) Immunofluorescence of wt HaCaT cells stained with antibodies against METTL16 (green) and fibrillarin (red) or transfected with plasmid encoding GFP-tagged UBF1/2 (green) and stained with antibody against METTL16 (red). DNA was stained with DAPI (blue). The fluorescence intensity, plotted along the white line that runs through the cell nucleolus, was calculated using Leica software. Scale bars, 5 μm. (**B**) Co-IP of METTL16 and Western blot analysis of METT16, NOLC1, TCOF, UBF1/2, and fibrillarin, studied in wt MEFs and LMNA dn cells. (**C**) A representative image of RIP-qPCR assay for the abundance of m^6^A in rRNA is shown. The highest m^6^A positivity was observed in 18S rRNA of UVC-irradiated LMNA dn cells. Data are shown as a percentage of input.

METTL16 has been previously reported to localize not only to the nucleoplasm but also to the nucleolus [5]. Using an antibody against the METTL16 protein, we showed METTL16 in the nucleoli of all cell lines studied (Figure 1A,C). METTL16 did not colocalize with UBF1/2 proteins (specific components of the transcription of ribosomal genes), which mainly occupy the border-line between Fibrillar Center (FC) and Dense Fibrillar Components (DFC) of the nucleolus [27]. However, there was partial colocalization between METTL16 and fibrillarin, which is a marker of DFC and occupies the nucleolar regions of ribosome maturation (Figure 6A). Since a high level of METTL16 was found in the nucleoli, we investigated the function of METTL16 in this nuclear compartment. Using coimmunoprecipitation, we explored whether the selected nucleolar proteins, including NOLC1, TCOF, UBF1/2, and fibrillarin, interact with METTL16 in wt and LMNA dn MEFs. We observed interactions between METTL16 and UBF1/2, METTL16 and TCOF or NOLC1 (Figure 6B). Importantly, we did not find an interaction between the METTL16 protein and nucleolar methyltransferase fibrillarin (Figure 6B). Observed interactions were not affected by UVA irradiation or lamin A deficiency.

In the next step, we performed quantitative RNA immunoprecipitation analysis (RIP-qPCR), which was used to examine the level of m^6^A in rRNA. These results showed a barely detectable m^6^A positivity in 28S rRNA (studied fragment was 151 bp), but there was a strong m^6^A positivity in 18S rRNA (studied fragment was 138 bp) in LMNA wt cells irradiated by UVC light that additionally upregulated METTL16 (Figure 4F and Figure 6C). A low level of m^6^A in 18S rRNA was observed in Lap2α dn cells, which were characterized by a very low level of METTL16 (Figure 5D and Figure 6C).

### 3.6. METTL16 Interacts with Lamin B Receptor

**Figure 7:**
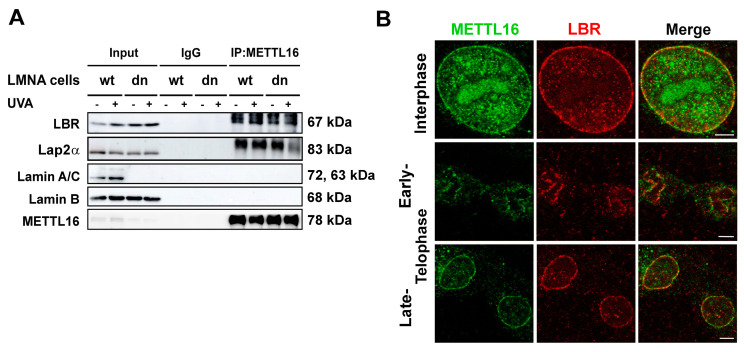
Interaction between METTL16 and lamins or lamin-associated proteins. (**A**) Co-IP of METTL16 with LBR, Lap2α, lamin A/C, and lamin B in wt MEFs and LMNA dn cells. (**B**) Immunofluorescence of MEFs stained with antibodies against METTL16 (green) and lamin B receptor (LBR, red). Scale bar, 5 μm.

To further investigate the function of METTL16 and its connection to lamins, we coimmunoprecipitated METTL16 and assessed the interaction of METTL16 with lamins A/C, B-type lamins, lamin B receptor (LBR), and Lap2α (Figure 7A,B). The LBR protein was selected due to its high density close to the inner nuclear membrane, similarly to that observed for METTL16 decorating the nuclear lamina. Moreover, both proteins appeared at the nuclear lamina at the same cell cycle stage, in telophase (Figure 7B). Because A-type lamin depletion affected the pool of METTL16, Lap2α, as a lamin A binding partner, was also studied (Figure 7A). These experiments demonstrated a specific interaction between METTL16 and LBR or METTL16 and Lap2α. UVA irradiation and lamin A deficiency showed no effect on protein-protein interactions. On the other hand, we observed no interaction between METTL16 and B-type lamin, or METTL16 and lamins A/C (Figure 7A,B).

## 4. Discussion

It is well-known that METTL16 (syn. METT10D) is an RNA methyltransferase installing m^6^A on U6 small nuclear RNA (U6 snRNA) and S-adenosylmethionine (SAM) synthetase pre-mRNA. Moreover, SAM homeostasis is important for regulating METTL16 function, and vice versa, which contributes to mRNA stability, splicing, while also promoting of translation via the function of m^6^A in RNA [10,27,28,29]. METTL16 also installs m^6^A on intronic polyadenylation (IPA) sites, which confirms the role of METTL16 in the splicing process [30]. However, a proper biological function of both METTL16 as a “writer” and m^6^A RNA as epitranscriptomic features must be specified, especially in cancer cells and during DNA repair processes. For example, it was revealed that a poor prognosis of colorectal cancers is associated with a high level of m^6^A RNA, and in these cells, there is an overexpression of METTL3, METTL16, and WTAP regulatory proteins [31,32]. We have recently shown distinctions between METTL3/METTL14 and METTL16 proteins in embryonic stem cells (ESCs) experimentally stimulated into cardiomyocytes. We have also observed that the nuclear pools and nuclear distribution of METTL3/METTL14 proteins in mouse ESCs were different from those studied in METTL16 [33]. Ruszkowska et al. [11] also showed that the molecular structure of METTL16 is different from the structure of METTL3 and METTL14 proteins. Moreover, in mouse 16-cell embryos, characterized by Mettl16 depletion, it was found that the mRNA level was reduced. This observation indicates that METTL16 plays a role in early development and, therefore, cell differentiation [17,33].

m^6^A in RNA was also found to be functional in UVA-induced DNA lesions [8,34]. Additionally, Svobodova Kovarikova et al. [19] showed that METTL16 methyltransferase is likely responsible for maintaining the high level of m^6^A RNA in locally microirradiated chromatin. We indirectly confirmed these results by observing that UVA and UVC irradiation upregulates METTL16 (Figure 4D,F and Figure 5B,C). A high m^6^A density in 18S rRNA, but not 28S rRNA was observed in UVC irradiated LMNA dn cells, characterized by a high level of METT16 (Figure 4F and Figure 6C). Our analyses suggest that the high level of METTL16 in the cells corresponds to a high level of m^6^A in 18S rRNA (Figure 4F, Figure 5D and Figure 6C), but additional METTL16 knock-out experiments should be performed.

In terms of the nuclear localization profile, it was observed by Nance DJ et al. [26] that METTL16 occupies not only the cell nucleus but also the cytoplasm. These authors suggested that METTL16 is also a cytoplasmic methyltransferase that works distinctly in the nucleus and the cytoplasm. Nucleo/cytoplasmic distribution of METTL16 may be cell type-specific. Also, it is unclear whether METTL16-specific target(s) is (are) identical in the nucleus and the cytoplasm [26]. Here, we show that the nuclear localization of METTL16 is cell type- and cell cycle-specific. Similarly, considerable changes in subcellular localization and cell-type specificity were observed by Herrmann et al. [35] for arginine methyltransferases (PRMTs). These authors showed a distinct Nucleo/Cytoplasmic ratio for PRMT1, PRMT2, PRMT3, and PRMT4. Interestingly, in breast cancer cells MCF7, a high density of PRMT1 was found inside the cell nucleus, while osteosarcoma U2OS cells were characterized by PRMT1 localization in the cytoplasm. In general, these data document a cell type-specific nuclear distribution of methyltransferases (Figure 1A–C, and [35]; https://www.proteinatlas.org/ENSG00000127804-METTL16/cell; accessed on 7 July 2021).

Here, we show that the nucleolus is a prominent site of METTL16 function, which can be linked to the N6-adenosine methylation in ribosomal RNA. Due to the high level of METTL16 that we observed in the nucleolus, we assumed that METTL16 could potentially interact with other nucleolar components. The interaction of METTL16 with nucleolar components such as NPM, DHX9, ILF3, and ILF2, mostly in an RNA-independent manner, was demonstrated by Brown et al. [14]. Our results showed the interaction between METTL16 and UBF1/2, TCOF, or NOLC1. Significantly, these protein-protein interactions were not affected by LMNA deficiency or by UVA irradiation (Figure 6B).

High METTL16 abundancy was also observed on the nuclear membrane; however, it is unclear whether METTL16 plays a role in nuclear lamina stabilization. We found no interaction between A-type or B-type lamins and METTL16, but there was an interaction between METTL16 and lamin B receptor or METTL16 and Lap2α (Figure 7A,B). Zhang et al. [36] documented that lamin A stabilizes nuclear speckles that are the reservoirs of splicing factors and also sites of epigenetically essential methyltransferases METTL3 and METTL14. However, here, we did not observe a direct link between the METTL16 protein and nuclear lamins.

In summary, we found a high density of the METTL16 protein in the nucleolus and the nuclear periphery of mouse cells, but METTL16 does not interact with nuclear lamins, despite the fact that A-type lamin depletion and Lap2α deficiency led to METLL16 downregulation. Conversely, we observed a pronounced interaction between METTL16 and lamin-associated proteins. Our additional contribution to the knowledge on the METTL16 function is that in the cell nucleus, there is cell cycle-specific distribution of METTL16, and a high level of METTL16 corresponds to a high density of m^6^A in 18S rRNA, but not 28S rRNA. Importantly, the METTL16 protein interacts with specific proteins of the nucleolus, including NOLC1, TCOF, and UBF1/2. We also, observed METTL16 interaction with DNA damage-related protein DDB2. Together, our data show that the METLL16 protein is multifunctional, likely regulating processes in the nucleolus and contributing to DNA damage response.

## Data Availability

Original micrographs (files in gigabytes, GB) are on-demand; please address Eva Bártová (e-mail: bartova@ibp.cz) or Lenka Stixová (e-mail: lenka@ibp.cz).

## Supplementary Materials

The following are available online at https://www.mdpi.com/article/10.3390/life11070669/s1, Figure S1: original membranes of western blots.
