# Supplementary figures and images for: Localization of METTL16 at the Nuclear Periphery and the Nucleolus Is Cell Cycle-Specific and METTL16 Interacts with Several Nucleolar Proteins
Source: Life (Basel). 2021 Jul 8;11(7):669. doi: 10.3390/life11070669 (PMC8305168; doi:10.3390/life11070669)

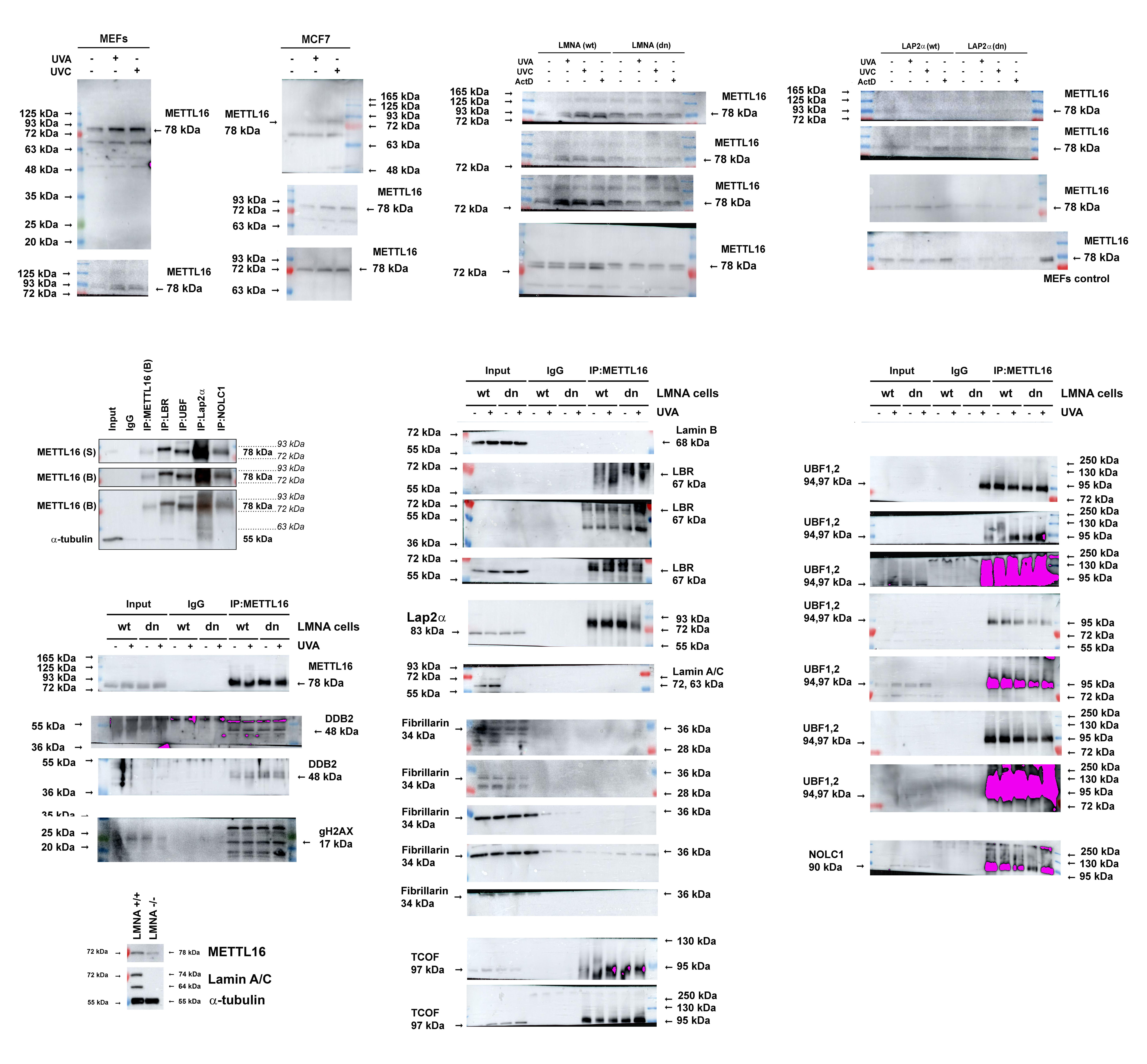

Supplement: Supplementary file 1 [file life-11-00669-s001.zip › life-1210405-SI.jpg]
